# Uncovering the link between inflammatory rheumatic diseases and male reproductive health: a perspective on male infertility and sexual dysfunction

**DOI:** 10.1007/s00296-024-05602-8

**Published:** 2024-05-02

**Authors:** Marlen Yessirkepov, Burhan Fatih Kocyigit, Kairat Zhakipbekov, Erbolat Adilbekov, Kassymkhan Sultanbekov, Mazlum Serdar Akaltun

**Affiliations:** 1 Department of Biology and Biochemistry, South Kazakhstan Medical Academy, Shymkent, Kazakhstan; 2 Department of Physical Medicine and Rehabilitation, University of Health Sciences, Adana City Research and Training Hospital, Adana, Türkiye Turkey; 3 Department of Organization and Management and Economics of Pharmacy and Clinical Pharmacy, Asfendiyarov Kazakh National Medical University, Almaty, Kazakhstan; 4 Central City Clinical Hospital, Almaty, Kazakhstan; 5 Department Social Health Insurance and Public Health, South Kazakhstan Medical Academy, Shymkent, Kazakhstan; 6 Faculty of Medicine, Department of Physical Medicine and Rehabilitaton, Gaziantep University, Gaziantep, Türkiye Turkey

**Keywords:** Male infertility, Male sterility, Rheumatic diseases, Rheumatism, Inflammation

## Abstract

Inflammatory rheumatic diseases (IRDs) refer to a range of persistent disorders that have a major influence on several physiological systems. Although there is much evidence connecting IRDs to sexual dysfunction and fertility problems, research specifically focusing on male infertility in relation to these diseases is sparse. This review addresses the complicated connection between IRDs and male infertility, emphasising the physiological, psychological, and pharmacological aspects that influence reproductive health outcomes in men with rheumatic conditions. We explore the effects of IRDs and their treatments on many facets of male reproductive well-being, encompassing sexual functionality, semen characteristics, and hormonal balance. Additionally, we present a comprehensive analysis of the present knowledge on the impact of several categories of anti-rheumatic drugs on male reproductive function. Although there is an increasing awareness of the need of addressing reproductive concerns in individuals IRDs, there is a noticeable lack of research especially dedicated to male infertility. Moving forward, more comprehensive research is needed to determine the prevalence, risk factors, and mechanisms driving reproductive difficulties in males with IRDs. We can better assist the reproductive health requirements of male IRD patients by expanding our understanding of male infertility in the setting of rheumatic disorders and implementing holistic methods to care.

## Introduction

Inflammatory rheumatic diseases (IRDs) encompass a variety of chronic conditions that have a major impact on multiple physiological systems. There is evidence suggesting that IRDs have an intricate link with sexual dysfunction and fertility [1, 2]. The decline in reproductive health cannot be exclusively attributed to the physical effects of chronic diseases and necessitates a comprehensive approach that considers psychosocial and cultural variables [3]. Curiously, there exists a disparity in the investigation of reproductive issues among patients with rheumatic disorders. While sexual health is examined equally in both genders, research on fertility and gonadal function is more commonly conducted in women [4, 5].

Infertility is defined as the failure to conceive after one year of sexual activity that is unprotected. It is thought to afflict approximately 15% of partners globally [6]. Male infertility can be categorized into two main types based on its cause: congenital, which includes conditions like Klinefelter syndrome, and acquired, which includes factors such as varicocele and accessory gland infestations. Semen analysis is regarded as the fundamental method for diagnosing potential male infertility. Nevertheless, a significant proportion of patients (30–50%) experience unexplained alterations in semen characteristics, and approximately 15% of these individuals remain infertile despite the absence of any observable abnormalities [7, 8].

**Fig. 1:**
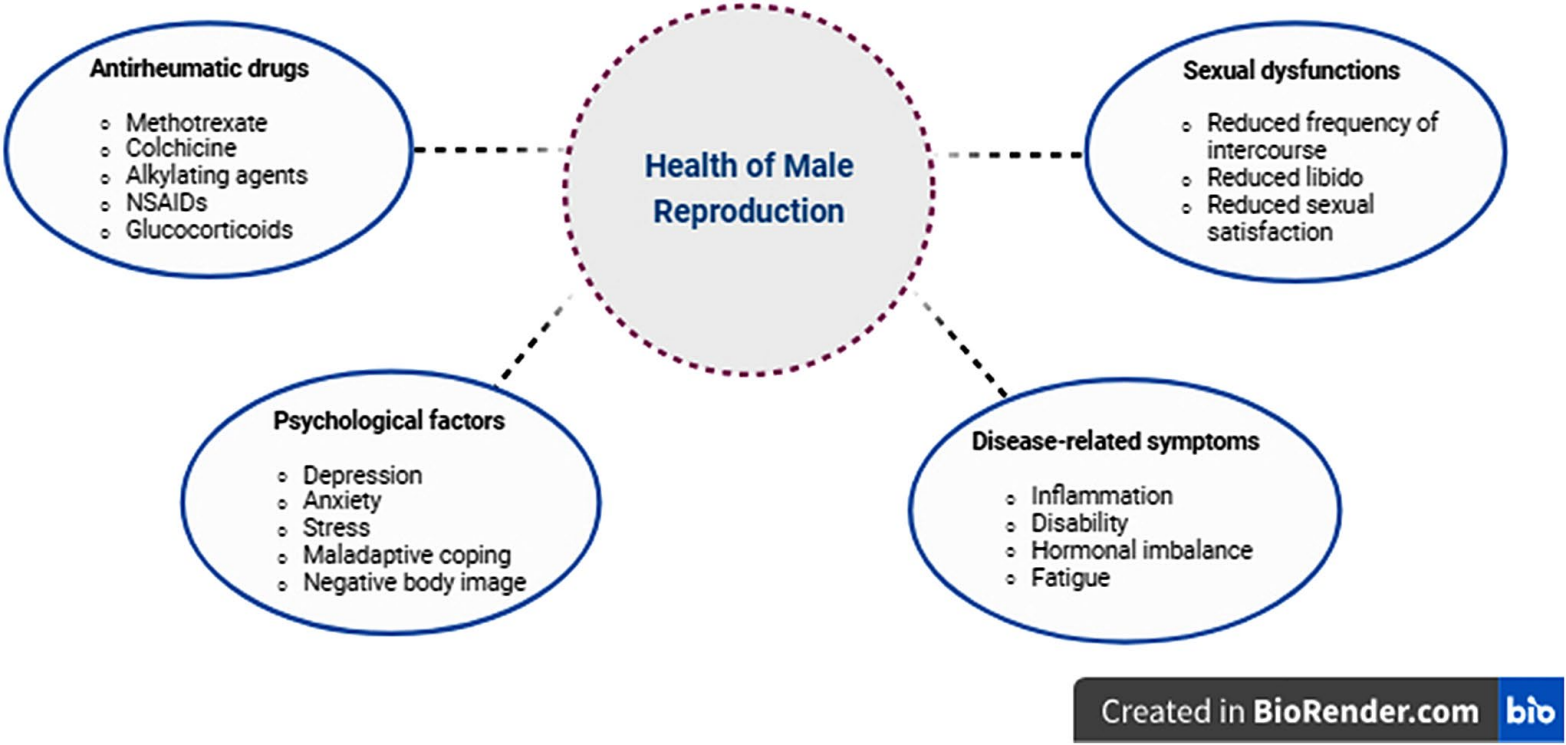
Links between inflammatory rheumatic diseases and male reproductive health

The influence of IRDs on sexual health and fertility is intricate when viewed from a male standpoint. The presence of IRDs and their medical care can significantly affect multiple facets of male reproductive health [9]. Stress caused by IRDs, physical ailments, emotional issues, and challenges with partnerships can lead to a decrease in sexual activity and satisfaction. The presence of chronic pain, tiredness, and low confidence might diminish an individual’s sexual desire, thus leading to a decrease in the quantity of sexual activity. The enjoyment of sexual contact may be impaired due to challenges in selecting poses that do not induce discomfort or pain [10, 11]. Chronic inflammation, as a result of IRDs, and the use of disease-modifying anti-rheumatic drugs (DMARDs) or immunosuppressant agents during care may influence semen quality [12]. This impact can occur by changing the activity of accessory glands, interacting with sperm transportation, and directly influencing the process of sperm production. Furthermore, IRDs are linked to both erection problems and hypogonadism [13, 14] (Fig. 1).

The emergence of reproductive rheumatology has contributed to the advancement of clinical care for patients with IRDs, establishing a specialist pregnancy center as part of routine rheumatic practices. These centers’ primary purpose is to focus on patient’s disease activity and how it affects sexual function-ability and fertility. Second, collaborate with the patient on the potential consequences of anti-rheumatic agents on pregnancy and birthing expectations [9].

This article aims to specifically address male infertility related to IRDs, which is a relatively neglected field. We aim to thoroughly analyze the subject by establishing separate subheadings for each disease category. Furthermore, we provide a concise overview of the association between anti-rheumatic drugs and fertility. Finally, we offer viable non-pharmacological methods for managing male fertility issues within the framework of IRDs.

## Search strategy

Relevant publications were obtained from Web of Science, Scopus, Medline/PubMed, Scopus, and DOAJ by searching for the terms “male infertility” or “male sterility”, and “rheumatic diseases” or “rheumatology” or “rheumatoid arthritis” or “ankylosing spondylitis” or “spondyloarthritis” or “systemic lupus erythematosus” or “Sjogren’s syndrome” or “systemic sclerosis” or “gout” or “myositis” or “Behcet disease” or “familial Mediterranean fever” or “systemic vasculitis” or “anti-rheumatic drug” or “rehabilitation”. Only English articles published prior to January 2024 were taken into account. No definitive timeframe was established. Furthermore, we conducted a comprehensive analysis of the references cited in the articles obtained using our search methodology and selected the ones that we considered relevant. The search approach was established in accordance with the conditions specified by Gasparyan et al. [15].

## Rheumatoid arthritis

Rheumatoid Arthritis (RA) is a chronic and multifactorial disease that impacts many people and can lead to progressive joint damage [16]. Epidemiologic studies show that RA occurs in approximately 0.5-1% of cases, and women are affected 2–3 times more often than men [17]. Although RA typically involves the joints, extra-articular involvement can also be seen during the disease. The disease can affect sex life for physical reasons such as fatigue, joint stiffness and swelling, pain, functional limitations, and psychological reasons. Body image issues and medications can also affect sexual function [18]. Gordon et al. [19] reported erectile dysfunction in 10 of 31 RA patients (33%) and decreased libido in 15 patients (50%). Nasr et al. [20] found erectile dysfunction in 11 (45.8%) of 24 male RA patients and 2 (11.1%) of 18 healthy controls. Miedany et al. [21] showed that 53.8% of male RA patients had sexual dysfunction in a multicenter study. In this study, age, cardiovascular disease, intramuscular methylprednisolone injection, fatigue, pain scores, and severity of disorder were found to be associated with erectile dysfunction. Similarly, Berlo et al. [22] showed in a multicenter study that sexual activity and libido were lower in male RA patients. Decreased hypothalamic-pituitary-gonadal axis activity has been reported in RA patients. Previous studies have shown that testosterone and luteinizing hormone (LH) levels are lower in men with RA, and this may suggest a central hypoandrogenism [23, 24]. Shiraishi et al. [25] reported that anti-sperm antibodies were detected in one of 32 RA patients, but there was not enough information to establish a relationship. Fertility and sexual function may be affected concerning joint pain, fatigue, cardiovascular disease, psychosocial status, deterioration of body image, and medications used. Hill et al. [26] observed in their study that 66% of RA patients had not previously been questioned about the effect of the disease on sexual life. Therefore, it is essential to determine the problems, to question sexual functions for treatment approaches, and to ensure good communication.

## Ankylosing spondylitis

Ankylosing Spondylitis (AS) is more common in men, especially in the reproductive period. There are many studies on sexual function in men with AS. Rostom et al. [27] found that 70 (95.9%) patients had not been asked about sexual activity before. Of the patients in this study, 30 (41%) had erectile dysfunction, and 28 (38.4%) had orgasm problems. Hip involvement, pain, sleep disturbance, and depression are possible causes that may affect sexual life in AS.

Varicocele is a condition where the veins in the pampiniform plexus become abnormally dilated. It is one of the basic mechanisms underlying treatable male infertility. Varicocele occurs in 35% of primary infertile men and up to 80% of secondary infertile men [28]. Studies have shown that the frequency of varicocele increases in AS patients compared to the healthy population. Due to inflammatory back pain, the valsalva maneuver is used more frequently. This maneuver may cause varicocele formation by increasing intra-abdominal pressure. Sperm abnormalities caused by varicocele may lead to infertility in patients with AS [29–31].

There are conflicting results in studies on sperm quality in AS patients. Ramonda et al. [32] found that motile sperm rate and testosterone levels were lower in AS patients, while LH and FSH levels were higher. Sperm movement was linked to disease activity. After one year of anti-tumor necrosis factor (anti-TNF) α treatment, sperm aneuploidies went down and hormone levels returned to normal. Interestingly, two studies found no significant difference in sperm quality between AS patients and healthy controls [33, 34]. Ozgocmen et al. [35] observed decreased sperm concentration, sperm motility, and impaired morphology in untreated patients. It was reported that this deterioration may be related to increased superoxide dismutase levels in the seminal vesicle and increased oxidative stress. Chatzimeletiou et al. [36] found no difference in motility, concentration, DNA fragmentation, and aneuploidy incidence, while electron microscopy images showed a higher rate of morphological abnormalities in sperm. In addition to anomalies in the head, neck, and tail of the sperm, the presence of immature sperm and phagocytes was also revealed. Uzunaslan et al. [37] observed that male patients with AS had decreased fertility after diagnosis and had fewer children than healthy controls.

Ultimately, it is crucial for healthcare practitioners to recognise that sexual dysfunction is a significant outcome of AS. Therefore, they should actively address the sexual issues faced by AS patients by offering relevant information or making appropriate referrals to specialists. There is a lack of consensus on the issue of male infertility in patients with AS, and it is imperative to conduct rigorous investigations to determine the presence of male infertility and its related factors in AS patients.

## Systemic lupus erythematosus

Systemic lupus erythematosus (SLE) affects the skin, joints, kidneys, and heart and is more frequent among reproductive-age women [38]. Additionally, SLE can be seen in men and at any age. It generally has a more severe course in men [39]. Infertility and sexual dysfunction in men with SLE are relatively overlooked issues. The gonadal function may be affected in men with SLE. Hypogonadism is characterized by reduced testosterone and/or sperm production in the testes. Soares et al. [40] found lower sperm volume, total sperm count, sperm motility, sperm concentration, and more significant abnormalities in sperm morphology in men with SLE. In addition, testicular volume was found to be less in men with SLE, and the rate of having children after the diagnosis of the disease was significantly lower than in controls. Another remarkable point obtained from the study was that testicular volume was lower in patients who received intravenous cyclophosphamide treatment and had high FSH levels. Suehiro et al. [41] investigated Sertoli cell functions in male patients with SLE. They found that inhibin B levels, which indicate Sertoli cell functions and are a good indicator of gonadal functions in men, were low in men with SLE. They also showed that inhibin B levels were lower in those receiving intravenous cyclophosphamide, and sperm abnormalities were higher in those with low inhibin B levels. Another condition that may impair fertility in men with SLE may be anti-sperm antibodies. The frequency of anti-sperm antibodies in men with SLE was found to be 41%, and these antibodies are among the potential causes that may impair fertility in patients with SLE [41].

Renal involvement is more common in men with SLE compared to women. It is known that fertility and sexual functions may be impaired in men with renal failure. Therefore, it is thought that fertility may also be affected in men with lupus nephritis. In men with SLE with renal failure, fertility may be affected due to hormonal imbalances such as gonadotropin resistance and hyperprolactinemia. Erectile dysfunction may also be a significant problem in patients with lupus nephritis due to vascular involvement [42, 43]. Sex chromosome aneuploidies (abnormal number of sex chromosomes) associated with hypogonadism are more common in men with SLE. Dillon et al. [44] conducted in 316 male SLE patients, 3 Klinefelter karyotypes, 4 mosaic Klinefelter karyotypes, and 1 XX male karyotype were observed, while no sex chromosome aneuploidies were found in the control group.

The frequency of erectile dysfunction in men with SLE is higher than in healthy controls due to anxiety, depression, thrombosis, endothelial dysfunction, and medications [45, 46].

In conclusion, fertility and sexual function may be affected in male patients with SLE for various reasons. Considering that SLE affects the reproductive period, it is important to identify potential risk factors and determine treatment strategies with a multidisciplinary approach.

## Hyperuricemia and gout disease

Gout is a frequently occurring inflammatory condition marked by the accumulation of monosodium urate crystals [47]. Gout is associated with a higher prevalence of disorders such as obesity, coronary artery disease, hyperlipidemia, and persistent kidney conditions [48]. These comorbidities may affect sexual function in men with gout. Gout patients have higher rates of erectile dysfunction, according to several research [49]. One study showed that the prevalence of erectile dysfunction increased not only after the diagnosis of gout but also in the year before gout was diagnosed [50]. Uric acid is believed to be another contributing component to the development of erectile dysfunction. An elevation of 1 mg/dl in blood uric acid level has the potential to enhance the likelihood of experiencing erectile dysfunction by twofold [51]. Endothelial dysfunction has also been reported as a mechanism that may impair erectile function in hyperuricemia [52]. Although it is predicted that lowering uric acid levels in gout patients will have positive effects on sexual health, there are no studies investigating the effects of urate-lowering treatments on sexual health. In conclusion, erectile dysfunction is a common clinical condition in patients with gout, and clinicians should plan appropriate treatment strategies when necessary.

Ma et al. [53] found that male individuals with hyperuricemia had lower semen volume and total sperm count. It was shown that semen volume and total sperm concentration decreased as serum uric acid levels increased. These results may be related to increased oxidative stress caused by hyperuricemia, endothelial dysfunction, decreased testosterone levels, and decreased seminal vesicle and epididymal secretory properties. In a study investigating the effect of hyperuricemia on in vitro fertilization-embryo transfer results, the biochemical pregnancy loss rate was significantly higher in couples with male hyperuricemia. Male serum uric acid level was positively correlated with biochemical pregnancy loss rate and negatively correlated with total fertilization rate [54]. The prevalence of hyperuricemia is rapidly increasing due to changing lifestyle and dietary habits. Therefore, it should be kept in mind that hyperuricemia may cause male infertility. Men with hyperuricemia should be informed about this issue, and lifestyle changes, diet, and urate-lowering interventions should be planned if necessary.

## Systemic sclerosis

Deposition of extracellular matrix in several structures is a hallmark of systemic sclerosis (SSc), a chronic, progressive condition [55]. Vascular injury, endothelial damage, and microangiopathic dysfunction can lead to vascular complications. An often-seen consequence of vascular disease is erectile dysfunction. According to numerous research, SSc patients have an erectile dysfunction prevalence of over 80% [56–58]. This condition is not thought to be related to hormonal influence [59]. Endothelial dysfunction in SSc causes a decrease in nitric oxide release. Decreased nitric oxide levels may cause insufficient cyclic guanosine monophosphate formation and lead to erectile dysfunction. Decreased penile blood flow and fibrotic changes in the tunica albuginea and corpora cavernosa may also be considered as possible causes of erectile dysfunction [60].

Sirithanaphol et al. [58] In their study of Thai men, they found that phimosis was also a common genital abnormality in SSc. Although there are many causes of phimosis, 13.3% of patients with SSc had phimosis, higher than the average population. Similar to the expected skin tension in SSc, it may cause phimosis by creating tension in the penile skin.

In a study conducted on SSc patients, the frequency of primary hypogonadism was found to be 23.3%. Although the frequency of hypogonadism was high in the study, since there was no control group, it cannot be concluded that the frequency of primary hypogonadism increased compared to healthy controls. In this study, risk factors for primary hypogonadism were found to be late-onset disease, late initiation of corticosteroid treatment, and hypertriglyceridemia [61].

In conclusion, erectile dysfunction should be considered when evaluating SSc patients. There are no studies on male fertility in patients with SSc. Considering the drugs used in the treatment of SSc and the etiopathogenesis of the disease, there are question marks on whether fertility is affected. Therefore, physicians planning treatment should be aware of reproductive health and plan diagnostic evaluations and interventions in patients with risk factors. Studies on male infertility in SSc patients are needed to fill the gap in the literature.

## Idiopathic inflammatory myopathies

Idiopathic inflammatory myopathies (IIM) refer to a diverse collection of inflammatory muscle disorders that result in muscle weakening and potential involvement of internal organs [62]. Since dermatomyositis (DM) is a rare rheumatologic disease, data are limited. There are very few studies on sexual function in patients with dermatomyositis. In a study of 5 post-pubertal male juvenile DM (JDM) patients, FSH, LH, prolactin, total testosterone, TSH, and free T4 values were normal in all patients. Teratozoospermia (abnormal sperm morphology) was present in all JDM patients but in 4 (80%) of controls. The prevalence of anti-sperm antibodies was comparable in both groups. Every JDM patient exhibited mild abnormalities in the morphology of their sperm, specifically in the head, midsection, and/or tail of the spermatozoa [63]. .

In a study evaluating twenty-five male IIM patients, the frequency of sexual activity and spontaneous pregnancy rate were significantly lower in IIM patients. Problems such as decreased sperm count, decreased sperm motility, and abnormal morphologic changes are more common in IIM patients [64]. The frequency of erectile dysfunction is also higher in male patients with IIM than in healthy controls [65]. As a result, reproductive health is impaired in men with IIM. Possible reasons for this include muscle weakness, physical inactivity, fatigue, and depression. Therefore, clinicians should evaluate fertility and sexual functions while evaluating patients with IIM and provide necessary information to the patients.

## Sjögren’s syndrome

Sjögren’s Syndrome (SS) is characterized by inflammation of the salivary and lacrimal glands, leading to mucosal dryness with decreased exocrine secretions. In addition to exocrine gland involvement, extra-glandular involvement, such as interstitial nephritis, interstitial lung disease, and neuropathy, may also be present. The etiology of SS is not known precisely. Genetic factors, environmental factors, and hormonal causes are thought to play a role in developing the disease. SS is more common in women, with a ratio of 10–20:1 [66]. Although problems such as vaginal dryness and dyspareunia in female SS patients have been shown in many studies, there are very few studies on sexual functions and fertility in male SS patients [10]. Margiana et al. [67] reported that the frequency of erectile dysfunction may be increased in men with SS, but the underlying mechanisms are not precise.

Due to increased rates of depression, autonomic neuropathy, and endothelial dysfunction, erectile dysfunction may be expected in patients with SS, and fertility may be affected. There are not enough studies on fertility and reproductive health in men with SS, and high-quality studies should be conducted on this subject.

## Behcet disease

Behcet disease (BD) is a chronic, multisystemic inflammatory disease that causes recurring oral and genital ulcers, skin lesions, and uveitis [68]. Although significant attention has been dedicated to the well-known signs of BD, the impact of this disorder on male reproductive health, mainly male infertility, remains a largely unexplored area of research [69].

Varicocele is characterized by the abnormal enlargement and twisting of the veins in the pampiniform plexus, and it is commonly linked to elevated male infertility [70]. Yilmaz et al. [71] assessed patients with BD for varicocele and epididymitis. The results indicate that BD patients had a higher frequency of varicocele and epididymitis. Varicocele patients exhibited a tremendous amount of genital ulceration and erythema nodosum lesions. No significant disparities were observed in smoking habits, epididymitis, arthritis, uveitis, or other medical variables while examining the distinction of varicocele in individuals with BD. Cetinel et al. [72] conducted a urological study on patients with BD over 2.5 years. They observed that the frequency of epididymitis during this time was 19.2%. The prevalence of epididymo-orchitis in BD varies based on the specific geographical region and the investigation’s design. There is a wide range of values, from 1.9% to about 30% [73, 74]. Men are more likely to be positive for HLA-B51/B5, which is linked to a higher occurrence of epididymo-orchitis [75].

While the exact mechanism remains uncertain, there appears to be a potential inclination towards male infertility in individuals with BD, as seen by the raised occurrence of varicocele, epididymitis, and orchitis. Nevertheless, some results contradict this notion. According to Uzunaslan et al. [37], there was no substantial rise in general infertility across BD patients. Furthermore, when male and female patients were assessed separately, there was no indication of a pattern of infertility for either gender. These findings indicate a necessity to prioritize BD and male infertility, to carry out high-quality research, and to attempt to clarify potential mechanisms.

## Familial Mediterranean fever

Familial Mediterranean fever (FMF) is a widely recognized genetic disorder that causes recurring fever and inflammation in the serous membranes. While the disease primarily impacts the Eastern Mediterranean region, sufferers are observed globally [1].

Male infertility in FMF is linked to physiological mechanisms associated with the disorder. Sudden orchitis with scrotal swelling, a relatively unusual sign of FMF that is frequently observed in pre-pubertal males, can cause testicular injury and disrupt spermatogenesis physiology. Testicular amyloidosis, although uncommon, can potentially contribute to the process [76]. Investigations into infertile male patients found azoospermia. The outcomes of testicular biopsy in patients with azoospermia have been interpreted as germinal cell aplasia with amyloidosis [77]. Certain studies have discovered a connection between male infertility and the use of colchicine, attributing it to the drug’s antimitotic characteristics [77, 78]. Colchicine tends to influence sperm movement, although this occurrence is infrequent when using the prescribed dosage in rheumatology practices [76].

Kaya Aksoy et al. [79] performed a study examining semen in adolescents with FMF. The findings indicate that a majority of adolescent patients with FMF exhibited atypical semen characteristics, predominantly characterized by reduced sperm motion.

Atas et al. [80] assessed the infertility of male and female patients with FMF. The findings indicated that 14.6% of females and 4% of males received a diagnosis of infertility. The analyses confirmed that the parameters linked to infertility were disease activity, disease initiation occurring below the age of 20, insufficient response to colchicine, and being female. The link between early FMF development and infertility may be attributed to the prolonged exposition of premature reproduction structures to the harmful impacts of the disorder. Elevated levels of inflammation and oxidative stress resulting from heightened disease activity may adversely affect reproductive health.

## Systemic vasculitis

Vasculitides are a group of systemic inflammatory disorders marked by uncontrolled inflammation of the vessels in the body. Various sizes of vessels can be impacted either separately or cumulatively. Vasculitides of various types can impact individuals throughout a wide age range, from children to older adults [81].

Polyarteritis nodosa is a necrotizing vasculitis that affects primarily medium-sized arteries [82]. Occasionally, orchitis may present as the initial manifestation of this condition. It is widely regarded as the most distinguishing clinical presentation of polyarteritis nodosa. Orchitis often affects only one testicle; the primary underlying cause is reduced blood flow in the testicular artery [83]. The clinical presentation described is not exclusive to polyarteritis nodosa, as comparable manifestations can also be observed in granulomatosis with polyangiitis or immunoglobulin A vasculitis [84]. Davenport et al. [85] documented the inclusion of the scrotum, penis, and testis in their case series on urologic system involvement in granulomatosis with polyangiitis. Testicular vasculitis is an uncommon condition. The testicular arteries can be affected by many types of systemic vasculitides. Brimo et al. [86] stated that testicular vasculitis can occur independently or coincidentally in some situations without being associated with a systemic condition. Many patients in their series of 19 cases were diagnosed with polyarteritis nodosa. Hernández-Rodríguez et al. [87] reported a collection of 72 cases of testicular vasculitis. Vasculitis affected the testis in 80.3% of cases, the epididymis in 44.6%, and the spermatic cord in 30.6%. Of the patients, 37 (51%) had only testicular involvement, while 35 (49%) had systemic vasculitis.

The findings above indicate that structures related to male reproductive health may be impacted as a component of the clinical range of vasculitis. One should take into account the potential for male infertility resulting from these impacts.

Common male infertility issues in rheumatic diseases are presented in Table 1.

**Table 1:** Overview of common male infertility issues in rheumatic diseases

Rheumatic disease	Potential male infertility and sexual dysfunction issues
Rheumatoid arthritis	• Erectile dysfunction • Decreased libido • Hypoandrogenism • Sexual life changes
Ankylosing spondylitis	• Erectile dysfunction • Orgasm problems • Increase in the frequency of varicocele • Sperm abnormalities
Systemic lupus erythematosus	• Hypogonadism • Sperm abnormalities • Hormonal imbalances • Erectile dysfunction
Gout disease	• Erectile dysfunction • Endothelial dysfunction related issues • Sperm and semen abnormalities • Issues associated with high uric acid levels
Systemic sclerosis	• Erectile dysfunction associated with vascular involvement • Endothelial dysfunction related issues • Decreased penile blood flow and fibrotic changes • Phimosis • Hypogonadism
Inflammatory myopathies	• Sperm abnormalities • Erectile dysfunction
Sjögren’s syndrome	• Erectile dysfunction
Behcet disease	• Increase in the frequency of varicocele and epididymitis
Familial Mediterranean fever	• Testicular injury and disrupt spermatogenesis physiology • Testicular amyloidosis • Sperm abnormalities
Systemic vasculitis	• Increase in the frequency of orchitis • Reduced blood flow in the testicular artery • Urologic system involvement • Testicular vasculitis

## Anti-rheumatic drugs and male infertility

Investigation into the potential link between anti-rheumatic drugs and male infertility is an emerging area of study. The effects of drugs on fertility can vary significantly across individuals and are influenced by multiple factors. These considerations may encompass the distinct attributes of the medication, dosage quantities, duration of use, and the individual’s preexisting health issues. Furthermore, the interplay among genetics, lifestyle variables, and overall health may influence the variations in individuals’ reproductive response to anti-rheumatic drugs.

### Methotrexate

Methotrexate (MTX) is a substance that inhibits the action of dihydrofolate reductase, an enzyme necessary for the production of DNA, RNA, and proteins [88]. It achieves this by acting as an antagonist to folic acid. Animal studies have demonstrated that it induces degeneration of spermatocytes, Leydig, and Sertoli cells in the testicular tissue of rats [89].

In human-centered studies, the results are contradictory. Ley et al. [90] compared patients on MTX treatment and healthy controls, focusing on assessing sperm quality and DNA integrity. While there were no discernible variations in basic sperm analysis across the groups, the researchers observed that the MTX group had compromised DNA integrity in sperm, causing harm by oxidative stress. Furthermore, there are articles providing evidence that MTX may have cytotoxic and genetic deleterious effects, potentially impairing the process of spermatogenesis and negatively impacting fertility [91]. The Swedish live birth registration study revealed no evidence of an elevated risk of congenital abnormalities, premature delivery, or small for gestational age in kids following paternal periconceptional exposure to MTX. Nevertheless, it was discovered that utilization of intracytoplasmic sperm injection substantially raised among males who were MTX exposed, indicating a potential decrease in fertility [92].

Conversely, Grosen et al. [93] observed that the utilization of low-dose mtx did not have an impact on the quality of sperm when compared to the controls. Additionally, the administration of MTX did not cause DNA fragmentation at the level of sperm.

Insufficient evidence exists about the impact of MTX on male fertility. The advice to discontinue MTX three months prior to pregnancy is considered safe. However, this recommendation depends mainly on the knowledge of the duration of spermatogenesis rather than an in-depth knowledge of the impact of MTX on spermatogenesis or its potential for causing congenital disabilities through paternal transmission [94].

### Leflunomide

There has been greater emphasis on paternal usage, and there is scant evidence about the connection between leflunomide and male infertility. Most publications describe cases of maternal exposure while barely mentioning paternal exposures. The baby was born healthy in one case report of paternal leflunomide exposure [95].

### Sulfasalazine

Sulfasalazine consists of 5-aminosalicylic acid and sulfapyridine. The chemical compounds of sulfapyridine can have detrimental effects on sperm, leading to oligospermia, impaired sperm motion, and an elevated likelihood of morphologically defective sperm [96]. Nevertheless, multiple studies have documented the reversal of detrimental effects on male fertility and the restoration of normal physiological functions following the cessation of sulfasalazine usage [97, 98]. Scientific data indicates that normal physiological function is restored three months following the cessation of drug usage [99].

### Hydroxychloroquine

There is a lack of specific research on the effects of hydroxychloroquine exposure in males, and no negative signs have been reported. Moreover, hydroxychloroquine is deemed congruent with maternal exposure and is, therefore, expected to be congruent with paternal input. Professional groups recommend that men who desire to conceive can safely continue taking this drug, as it is not associated with any potential harm [100].

### Non-steroidal anti-inflammatory drugs

No research was found investigating the effects of non-steroidal anti-inflammatory medications (NSAIDs) on inflammatory rheumatic or immune-mediated conditions. The investigations were carried out on participants who were generally in good health. Consequently, it may not be suitable to generalize the findings to rheumatic disorders. An investigation on naproxen revealed no substantial impact on the density and motility of sperm [101]. A study investigating the effects of ibuprofen revealed that this medication induces alterations to the hormonal system by selectively inhibiting the process of gene transcription in the human testes, resulting in a condition known as compensated hypogonadism [102].

### Systemic corticosteroids

Martens et al. [103] assessed the levels of testosterone, follicle-stimulating hormone (FSH), and luteinizing hormone (LH) in individuals with both long-term and active RA, as well as in healthy male controls. In comparison to healthy individuals, patients with RA who did not receive steroids exhibited typical amounts of testosterone but notably raised levels of FSH and LH. Furthermore, the group that received steroids exhibited decreased testosterone levels, along with the effects above. An analysis of Danish data examining birth outcomes of men who had steroid administration during pregnancy revealed no substantial rise in the likelihood of preterm delivery, small gestational age, or birth defects [104].

While high-dose corticosteroids can interfere with the hypothalamic-pituitary-gonadal axis and reduce the level of testosterone, there is not enough data to suggest that these drugs have an impact on male fertility [105].

### Colchicine

Colchicine, a commonly employed medication in rheumatology, possesses anti-inflammatory characteristics. Colchicine induces antimitotic properties by binding to tubulin and inhibiting microtubule polymerization [106]. In a study of male FMF patients, Kaya Aksoy et al. [79] observed that colchicine diminished sperm movement. According to Ben-Chetrit et al. [107], the movement of microtubules is critical for sperm motility. Therefore, it was proposed that treatment with colchicine would have a detrimental influence on sperm motility, contingent upon the length and concentration level. There have been recorded instances of azoospermia linked to colchicine [108]. Colchicine exposure in individuals with BD leads to abnormal sperm analysis [109]. Nevertheless, particular research examining the detrimental impacts of colchicine therapy on sperm has not yielded definitive results. In disorders marked by inflammatory activity, such as FMF or BD, it remains uncertain whether the decline in sperm parameters is mainly triggered by the treatment or the underlying disease itself [78].

### Cyclophosphamide

Cyclophosphamide is classified as an alkylating agent and can potentially induce irreversible or long-lasting oligospermia. Doses of cyclophosphamide exceeding 7.5 g/m^2^ provide an immense threat, resulting in permanent azoospermia in 90% of users [105]. Furthermore, abundant research indicates that cyclophosphamide cycles have a distinct detrimental impact on the condition of sperm and fertility hormones, mostly resulting in reduced sperm count and elevated FSH concentration [110, 111]. Male patients experiencing cyclophosphamide therapy should receive counseling regarding preserving fertility approaches. Cryopreserving semen for future use with technology for assisted reproduction is the most efficient method for preserving fertility in men. Preferably, semen should be obtained prior to cyclophosphamide administration or at least three months following the most recent dosage [105].

### Azathioprine

Most studies provided reassurance on the impact of azathioprine on male fertility [37, 112, 113]. Nevertheless, a cohort investigation indicated a tendency toward increased rates of infertility in patients who were administered azathioprine. However, the occurrence of infertility did not achieve statistical significance when compared to individuals who were not exposed to the drug [114]. Current evidence does not suggest an association between azathioprine and male infertility.

#### Biologic drugs

**Fig. 2:**
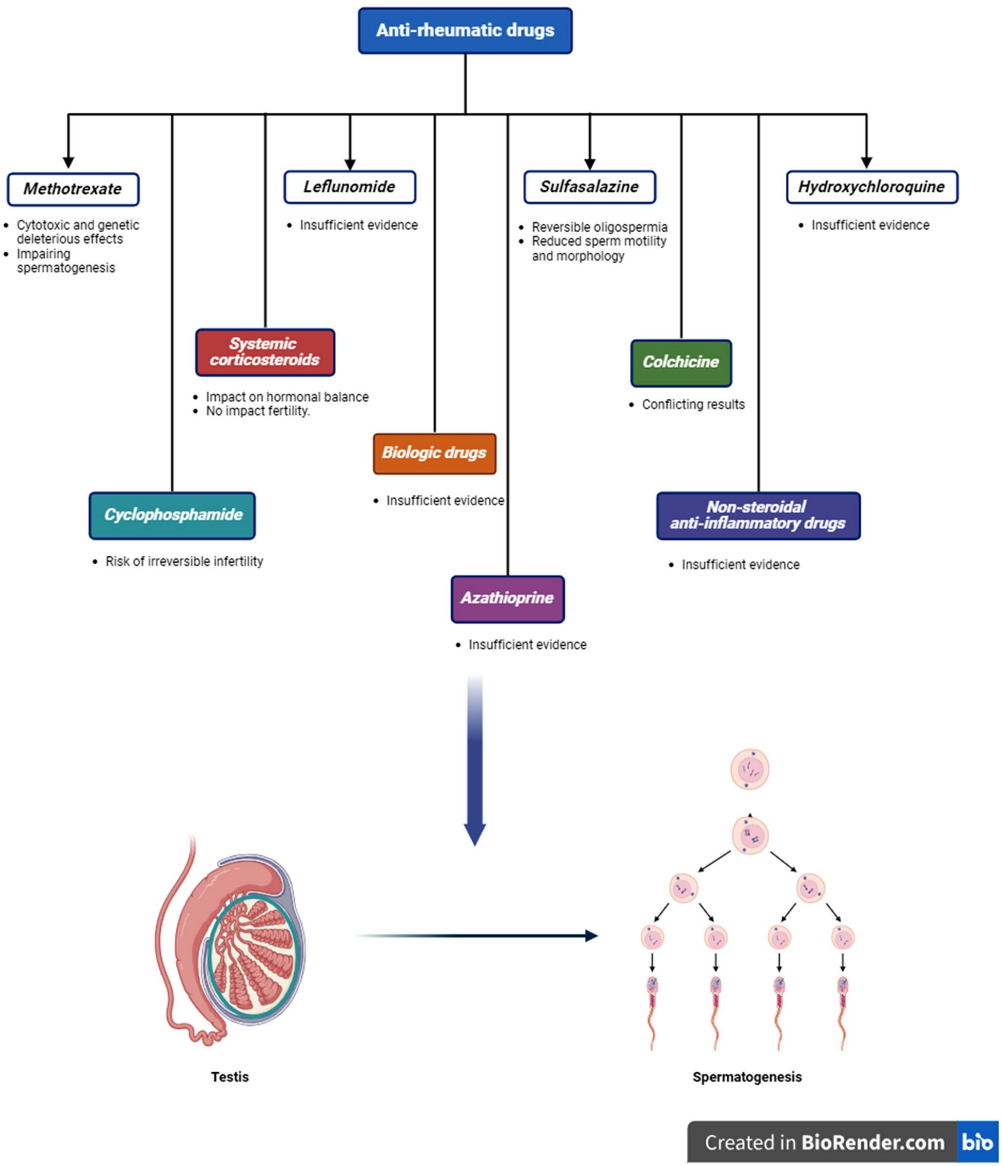
Effects of anti-rheumatic drugs on male fertility

Anti-TNF agents specifically target TNF-a and have demonstrated efficacy in treating several immune-related inflammatory disorders. This group of drugs has experienced growing utilization across several fields, particularly in rheumatology [115]. Most studies do not find evidence to establish a link between anti-TNF drugs and male infertility [116]. Despite a few research that has reported contradictory outcomes, the prominence of these papers is diminished due to their small sample size and methodological shortcomings [117, 118]. Furthermore, studies indicate positive fertility outcomes among male patients due to the notable reduction of inflammation through anti-TNF drugs [32, 119] (Fig. 2).

Human studies on the impact of rituximab on male fertility are limited despite positive findings in animal experiments [120, 121]. The evidence does not establish a link between abatacept, anakinra, tocilizumab, tofacitinib, secukinumab, and male fertility [116].

### Other drugs

The limited available data indicates no impact of cyclosporine A and mycophenolate mofetil on male fertility [116].

## Non-pharmacologic approaches for male infertility management

### Nutritional therapy

Mounting data indicates that adopting healthy eating styles, habits, and patterns is linked to enhancements in several factors related to sperm, such as the amount and quality, mobility, form, and fragmentation of DNA [122, 123]. Nutritional and environmental variables are crucial in maintaining the proper functioning of the reproductive system. Aside from demographic and established lifestyle variables (covering age, tobacco use, and alcohol consumption), there is mounting evidence that nutritional considerations significantly influence the proper functioning of the reproductive system [122]. From this standpoint, researchers have explored different dietary adjustments to address male fertility issues. The combination of omega-3 fatty acids, specifically docosahexaenoic acid and eicosapentaenoic acid, enhanced the movement of sperm [124]. Coenzyme Q10, selenium, l-carnitine, and vitamins C, E, and D have been documented to yield favorable outcomes on both the characteristics of sperm and rates of pregnancy [125].

Nutritional therapy exhibits potential as a complementary method for handling male infertility. While certain nutrients and dietary habits have been linked to enhanced sperm quality and reproductive well-being, it is crucial to view nutritional interventions as a component of a comprehensive approach that includes lifestyle modifications and expert supervision. Customized strategies will yield greater advantages for individuals grappling with male infertility.

### Spa therapy

Spa therapy, a diverse range of therapeutic methods that utilize mineral-rich thermal waters, mud baths, and other spa amenities, has garnered interest due to its potential to enhance general health and well-being [126]. Stress is recognized to be a factor in causing hormonal imbalances and can have a detrimental impact on the generation of sperm. Spa therapy typically includes relaxation techniques, hydrotherapy, and mud packs, which aid in the reduction of stress [127]. Sufficient blood circulation is crucial for maintaining the well-being of the reproductive systems and facilitating sperm production. Spa therapy enhances blood circulation, hence potentially benefiting male fertility. Enhanced circulation additionally aids in the dissolution and elimination of pollutants from the body. Regular spa baths can restore the proper activity of the endocrine glands and the human autonomic nervous system [128, 129]. Removing toxins from the body is assumed to have a beneficial impact on reproductive health.

Although spa therapy is commonly linked to relaxation and well-being, its specific effects on male infertility necessitate additional scientific investigation. One should consider the broader context of individual health and preferences when considering potential benefits such as stress reduction, increased circulation, and detoxification.

### Massage therapy

Massage therapy, an old healing method, has become famous for its possible therapeutic impact on different aspects of health [130]. Therapeutic massage techniques are designed to induce relaxation, diminish the presence of stress hormones, and enhance overall well-being [131, 132]. It decreases the overall amount of stress. This approach may exert a beneficial impact on male reproductive capacity. Specific massage techniques, particularly deep tissue, and Swedish massage can enhance blood circulation [133, 134]. Particular massage techniques can influence hormone levels by stimulating the endocrine system [135].

**Fig. 3:**
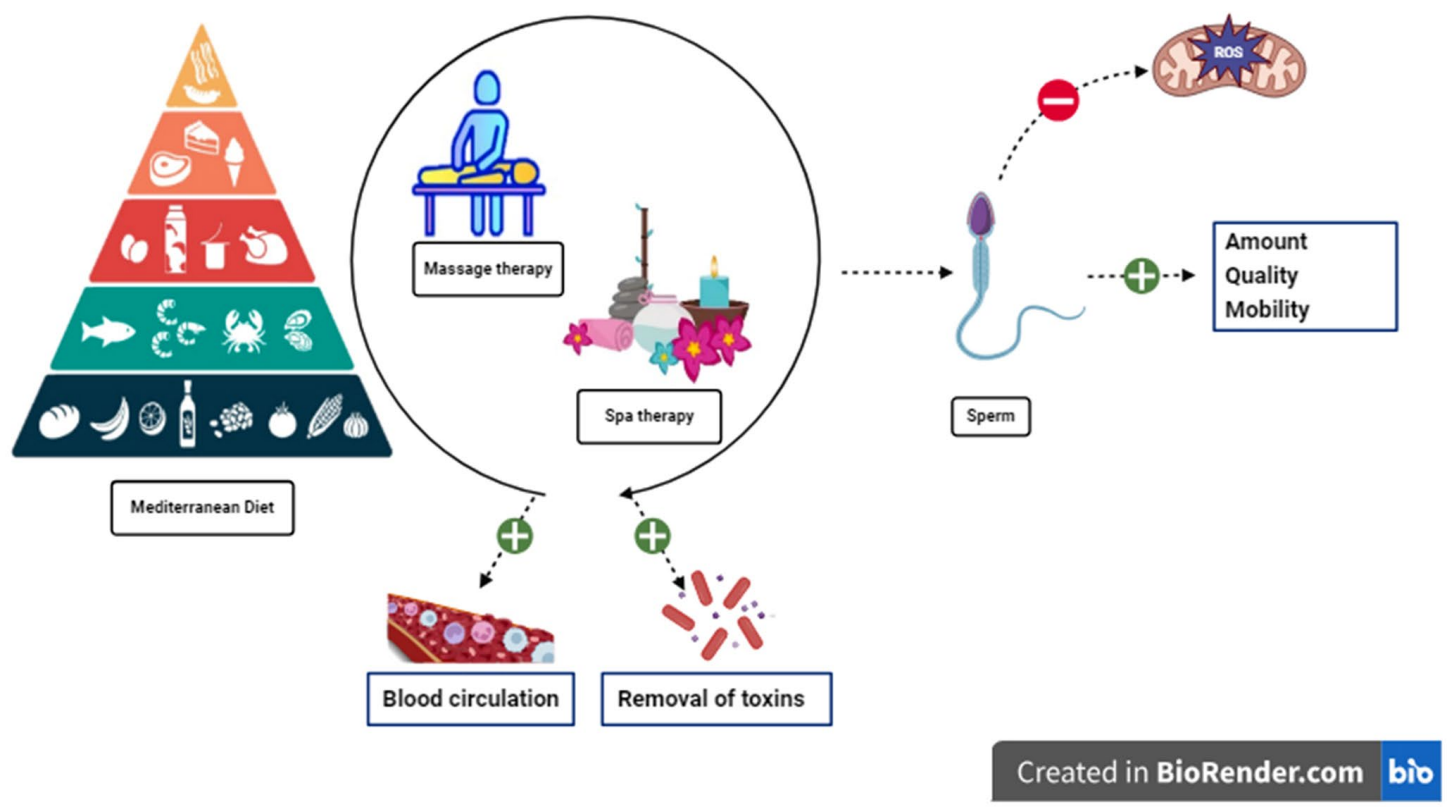
Visualization of nutritional, spa and massage therapies in male infertility

Furthermore, individuals with specific medical issues or anatomical abnormalities should consult with their healthcare providers prior to considering specialized massage therapy, in addition to the potential positive outcomes previously indicated. The optimal duration and frequency of massage therapy to achieve the most significant effect in managing male infertility has not been definitively established. Although there are a few anecdotal reports and a small number of studies indicating possible benefits, scientific data are scarce regarding the direct impact of massage treatment on male infertility (Fig. 3).

### Conclusion and future perspectives

The effects of IRDs on sexual health and fertility are complex, being influenced by both the clinical signs of the disorders and psychosocial variables. Although there is an increasing acknowledgment of the need of addressing reproductive concerns in individuals with IRDs, there is still a notable gap in research, specifically in the area of male infertility. Although sexual health is frequently investigated in both sexes, there is a greater emphasis on studying fertility and gonadal function in women, resulting in a relative lack of awareness regarding male-specific issues.

Current evidence indicates that IRDs and their treatments can impact various facets of male reproductive well-being. Chronic inflammation, physical restrictions, emotional stress, and the use of anti-rheumatic drugs are all factors that might contribute to fertility challenges among men with IRDs. Nevertheless, the precise mechanisms responsible for these effects have yet to be completely understood, and additional research is necessary to gain a deeper comprehension of the intricate interaction between inflammatory conditions, drugs, and male reproductive physiology.

Longitudinal investigations with larger sample sizes and rigorous methodology are required to determine the prevalence, risk factors, and mechanisms behind reproductive difficulties in males with different rheumatic diseases. Furthermore, investigation into the effect of certain anti-rheumatic drugs on male fertility is critical for guiding clinical practice and optimising treatment options.

Improvements in research and clinical practice in reproductive rheumatology will enhance our comprehension and management of the distinct requirements and difficulties encountered by males with IRDs in the future. Through promoting cooperation among researchers, medical professionals, and patients, we can strive to enhance the reproductive outcomes and overall well-being of those impacted by IRDs.
